# A computational survey of candidate exonic splicing enhancer motifs in the model plant *Arabidopsis thaliana*

**DOI:** 10.1186/1471-2105-8-159

**Published:** 2007-05-21

**Authors:** Mihaela Pertea, Stephen M Mount, Steven L Salzberg

**Affiliations:** 1 Center for Bioinformatics and Computational Biology, University of Maryland, College Park, MD 20742, USA; 2 Dept. of Cell Biology and Molecular Genetics, University of Maryland, College Park, MD 20742, USA

## Abstract

**Background:**

Algorithmic approaches to splice site prediction have relied mainly on the consensus patterns found at the boundaries between protein coding and non-coding regions. However exonic splicing enhancers have been shown to enhance the utilization of nearby splice sites.

**Results:**

We have developed a new computational technique to identify significantly conserved motifs involved in splice site regulation. First, 84 putative exonic splicing enhancer hexamers are identified in *Arabidopsis thaliana*. Then a Gibbs sampling program called ELPH was used to locate conserved motifs represented by these hexamers in exonic regions near splice sites in confirmed genes. Oligomers containing 35 of these motifs have been shown experimentally to induce significant inclusion of *A. thaliana *exons. Second, integration of our regulatory motifs into two different splice site recognition programs significantly improved the ability of the software to correctly predict splice sites in a large database of confirmed genes. We have released GeneSplicerESE, the improved splice site recognition code, as open source software.

**Conclusion:**

Our results show that the use of the ESE motifs consistently improves splice site prediction accuracy.

## Background

Alternative splicing is an important regulatory mechanism for many species, allowing them to generate multiple variant proteins from the same primary transcript. In order to predict the complete protein complement of any eukaryote, we need to detect alternative splice sites and put them together in the correct combinations. Algorithmic approaches to splice site prediction have relied mainly on the consensus patterns found at the boundaries between protein coding and non-coding regions [1]. However the sequence conservation found at the splice site junctions is not strong enough to accurately differentiate between introns and exons [2]. Additional sequences, residing at variable distances from splice sites, have been shown to function as *cis*-acting factor binding sites that regulate splicing either *in vivo *or *in vitro*. Although such splicing regulators have been identified in both exons and introns, exonic splicing regulators (ESRs) are generally better characterized, and are probably more common [3,4]. Such ESRs either enhance or suppress the utilization of both 5' and 3' splice sites. Much attention has been given to exonic splicing enhancers (ESEs) which promote the inclusion (as opposed to skipping) of the exons in which they reside. The first ESEs to be characterized were short, purine-rich motifs containing repeated GAR (GAA or GAG) trinucleotides, but subsequently many other sequences have been shown to have enhancer activity [5,6].

In animals, many exonic splicing enhancers are bound and activated by one or more of several related splicing factors known as SR proteins. The relationship between sequence-specific binding by SR proteins and the activation of splicing by exonic splicing enhancers is complex and incompletely understood. Although only a dozen or so splicing events have been shown to be enhancer-dependent, the existence of exonic splicing enhancers within constitutively spliced exons [7], the frequency of ESE motifs [8] and the absolute requirement for SR proteins by in-vitro splicing systems suggest that ESEs are ubiquitous, and required for all splicing events. It is estimated that as many as 15–20% of randomly appearing 20-mers contain a splicing enhancer [3] and computational methods have predicted hundreds of ESE motifs [9,10]. Thus, it appears likely that many sequences may act to affect splicing. What is clear is that the motifs recognized by SR proteins are short (8 or fewer nucleotides) and degenerate [6,11,12].

Several computational approaches have been undertaken to find the motifs characteristic of these splicing regulatory elements. In a recent study, Goren and colleagues [13] introduced a computational method that identifies ESRs based on conservation of wobble positions between orthologous human and mouse exons. Their method identified 285 putative ESRs, from which a sample of ten elements were shown experimentally to induce different levels of regulatory effects on alternative splicing. RESCUE-ESE, another computational approach, identifies potential ESEs based on the theory that exons with weak splice sites are more likely to require ESE activity for splicing [9]. The original study identified 283 exonic hexamers that were significantly enriched both in human exons relative to introns and in exons with weak splice sites relative to exons with strong splice sites; *in vivo *tests of these hexamers confirmed ESE activity. In another study, Zhang and Chasin [10] predicted human ESR motifs by comparing the frequency of 8-mers in internal noncoding exons versus unspliced pseudo exons and 5' UTRs of transcripts of intronless genes.

Previous computational work on detecting ESEs has focused almost exclusively on mammalian species. There are compelling reasons to believe that ESEs play an important role in plants as well. Early research on plant pre-mRNA splicing emphasized the role of AU-rich or U-rich sequences within introns [14,15]. These U-rich sequence elements play important roles in intron definition, and plants lack the very large introns that are associated with the need for exon definition [16]. On the other hand, a number of reports describe a role for exon sequences in the selection of plant splice sites [17-19]. SR proteins, the mediators of ESE activity in vertebrates, are highly conserved in plants [20,21]. This pattern of conservation includes reactivity with the monoclonal antibody mAb104 [22] and extends to function. A mixture of Arabidopsis SR proteins [23], and atRSZp22 in particular [24] can complement SR-deficient mammalian splicing extracts. Furthermore, plant SR proteins can influence splice site choice in mammalian nuclear extracts [25], and can regulate alternative splicing *in planta *[26,27].

The focus of this study is a new computational approach to identifying ESE motifs in the model plant *Arabidopsis thaliana*, and their use in improving splice site prediction accuracy. First we apply a similar approach to RESCUE-ESE to identify putative ESE hexamers in the flanking ends of a large set of known internal exons from *Arabidopsis*. Then we use a Gibbs sampling program called ELPH to identify statistically conserved motifs representing these hexamers in our data. In the end we show how these motifs can be used to improve splice site prediction. A significant improvement in specificity is obtained by incorporating the hexamer motifs into two leading splice site prediction programs, GeneSplicer [28] and SpliceMachine [29].

## Results and discussion

### Data sets

Our ESE analyses were done on several high-confidence Arabidopsis data sets. The first set, ESEAra, was extracted from a set of very high-quality gene models obtained from 5000 full-length transcripts sequenced released in 2001 [30] (These sequences are at [31] and at GenBank as accession numbers AY084215–AY089214.) Because internal homology in the data set could influence the results, we refined this reference set of gene models by using BLAST [32] to perform pairwise alignments between all genes. Sequences that aligned for more than 80% of their length with a BLAST E-value of less than 10^-10 ^were removed. The resulting ESEAra set includes 4046 genes containing of 17410 coding exons with an average length of 194 base pairs (bp). ESE motifs were determined on this data set.

A second data set was used to evaluate the accuracy of SpliceMachine after introducing the ESE motifs found in ESEAra. This data set consists of the 1323 *A. thaliana *genes used previously in the evaluation of both GeneSplicer [28] and SpliceMachine [28,29]. We will refer to this data set as GSAra.

To test the accuracy of our splice site predictor outside the gene sequences, we collected one additional data set consisting only of intergenic regions situated between annotated *A. thaliana *genes. We used the highly curated, re-annotated *Arabidopsis *chromosome II sequence (available from [33]) and extracted regions located more than 500 nucleotides from any annotated genes. We called this data set INTAra.

### ESE motifs

**Figure 1:**
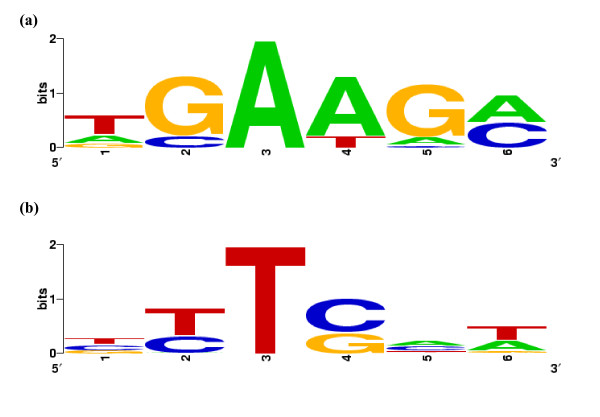
**Sequence logos for motifs detected in the ESEAra exons**. a) Motif detected at the 5'end of ESEAra exons, and b) motif detected at the 3'end of ESEAra exons. Both logos were computed with WebLogo [45].

We identified a total of 84 potential ESE elements in the flanking regions of exons in the ESEAra data set [see Methods]. Out of these 84 ESEs, 44 tend to be overly represented at the 5'end, 18 at the 3 'end and 22 at both ends (results shown in TableS1 [see Additional file 1]). The predicted ESE candidates contained the two hexamers TGAAGA and TGAAGC, which are equally strongly represented by the motif found by ELPH in the 5'end data, but they did not contain the consensus of the motif predicted in the 3'end data (see Figure 1). To find the motifs that were represented by these ESE hexamers we ran ELPH using each of the 66 5' ESEs and 40 3'ESEs as input seeds on the 5' and 3' flanking ends respectively of the internal exons in ESEAra. Running ELPH in this way generated position weight matrixes for all 84 input seeds but only 73 of the ELPH motifs found (62 at the 5'exonic ends and 30 at the 3'exonic ends) were significantly conserved in the data (P-value < 0.05).

ESE activity has been shown for several of the hexamers identified [34]. Out of the 84 hexamer motifs we identified as putative ESE elements, 35 (12 at the 5'end, 6 at the 3' and 17 at both ends) are included in a set of experimentally confirmed 9-mers that function as exonic splicing enhancers in *A. thaliana *(results shown in Table 1 and TableS1 [see Additional file 1]). Most significantly, for 8 of these 25 9-mers, mutation of one base (in one or two of our predicted ESE hexamers that are contained within that 9-mer) resulted in reduced ESE activity for the mutant ninemer (Table 1). It is also worth noting that the GAAGAA hexamer, the highest scoring ESE motif identified by our method, has long been known to function (as part of the 9-mer GAAGAAGAA) as an exon splicing enhancer in humans [35].

**Table 1:** Experimental evidence for predicted ESE hexamers.

9mer ESE	ESE Score	Mutant ESE	Mutant Score	Contained Hexamer Motifs
GAAGAAGAA	5	GCAGAAAAA	-1	gaagaa, aagaag
TGCTGCTGG	5			tgctgc, gctgct
TGCAGCTGG	5			gcagct, cagctg
GAAGATGGA	5			gaagat, aagatg, gatgga
GAAGGAAGA	5			gaagga, aaggaa, ggaaga
GAGAAGAAG	5			gagaag, gaagaa, aagaag
TTGGAGCAA	5			ttggag, ggagca
AGCTGCTGG	4			agctgc, gctgct
TGCTGGTGG	4			tggtgg
TGCTGCAGG	4			tgctgc, ctgcag
TGCTGCTCG	4			tgctgc, gctgct
TGCTGCTGC	4	TACTTCTGC	-3	tgctgc, gctgct
GAGGATTGA	4	GAGAATTGA	-1	gaggat
TGCAGATGA	4			gcagat, cagatg
CAAGAAACA	4			aagaaa
GAAGAGAAA	4	GCAGAAAAA	-1	aagaga
AAAGGAGAT	4			aaggag, aggaga, ggagat
GAAGAAAGA	4			gaagaa, aagaaa
GAGCAGAAG	4			gagcag
TGCTGCCGC	4			tgctgc
TTGAAGAAG	3	TTGAAAAAG	-3	ttgaag, tgaaga, gaagaa, aagaag
TTGAAGCTG	3	TTAAAGCTG	-3	ttgaag, tgaagc, gaagct, aagctg
GAAGATTGA	3	GAGAATTGA	-1	gaagat
TTTGGTGGA	3			tggtgg, ggtgga
ATGGAGAAA	3	ATTGAGAAA	-3	atggag, tggaga, ggagaa

Hexamer motifs that are contained within experimentally confirmed 9-mers with ESE activity (column 5). Experiments to confirm 9-mers are described elsewhere (S. Mount et al., manuscript in preparation). Column 1 shows the containing ESE ninemer, and column 3 shows ninemers without ESE activity, which are situated within 1–2 bp edit distance from the ESE ninemer. The ESE activity of each 9-mer in the table is shown by a score equal to log_2_(inclusion/skipping) [34].

### Splice site prediction

As mentioned above, several recent studies have described computational methods for identification of ESR elements. However few attempts have been made to improve splice site prediction by using these elements; one exception is a method for exon prediction that uses ESEs and ESSs [36]. One of the goals of our study was to provide a way to integrate the motifs predicted as potential ESEs into splice site prediction programs, in particular GeneSplicer. We used the 84 putative ESE motifs found by ELPH (66 for the 5'end and 40 for the 3'end, 22 of which appear at both ends) and the corresponding splice site score predicted by GeneSplicer as features in a linear support vector machine (LSVM). The LSVM created this way was integrated in the new splice site prediction system GeneSplicerESE.

**Figure 2:**
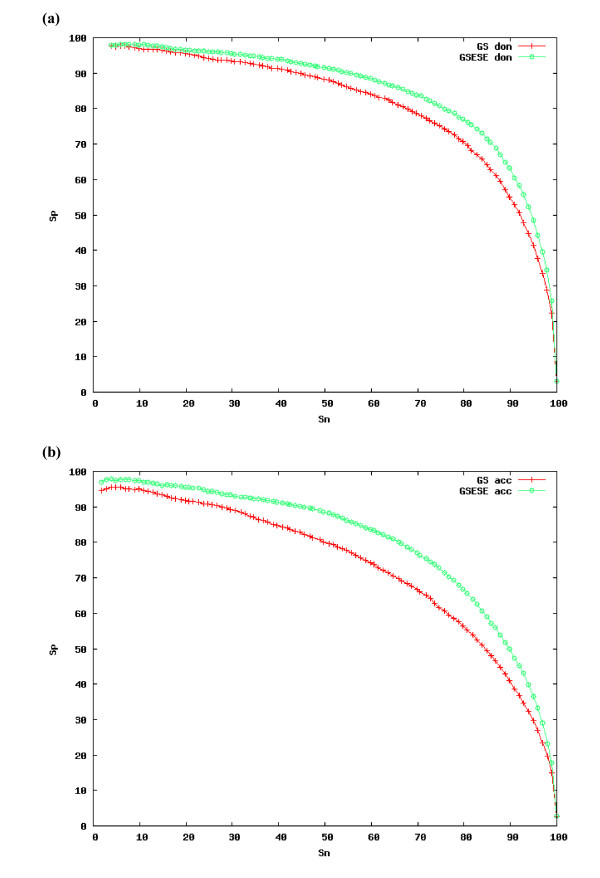
**Sensitivity versus specificity rates for GeneSplicer and GeneSplicerESE**. Sensitivity is defined as the fraction of all true splice sites found by the splice site predictor; specificity is the fraction of the predicted elements labelled correctly as splice sites. Rates are shown for a) donor sites (GS don and GSESE don), and b) acceptor sites (GS acc and GSESE acc). Results are obtained using a 5-fold cross-validation procedure on the ESEAra data set. Weight matrices for the selected motifs to describe each of the splice sites were recomputed on each training data set from the 5 partitions of the CV procedure.

To evaluate the splice site prediction accuracy of GeneSplicerESE, we applied a 5-fold cross-validation procedure on the ESEAra data set: the data were partitioned into 5 non-overlapping subsets, and each subset was held out separately while the system was trained on the remaining 4. Training included all positive examples, and 50,000 randomly selected negative examples. As negative examples we considered all dinucleotides in the ESEAra data set that matched the consensus splice site (AG for acceptors, and GT for donors), but did not overlap the confirmed splice sites. Accuracy was then measured on all positive and negative examples from the held out data. All motif position weight matrixes were recomputed on 50 bp flanking exonic sequences from the training data, but the length for the flanking sequence involved in equation (2) [see Methods] was chosen between 45 and 80 bp. The optimal length of this flanking region was chosen for each splice site by applying a 5-fold cross-validation procedure on the training data. Complete sensitivity vs. specificity plots for the original GeneSplicer and GeneSplicerESE on this data are shown in Figure 2. A significant increase in accuracy of GeneSplicerESE vs. GeneSplicer can be observed for both splice sites, with somewhat larger advantages occurring for acceptor sites. At the 95% sensitivity threshold (a threshold often used in splice site prediction), the false positive rate of GeneSplicerESE is 2.9% at the acceptor sites while GeneSplicer's false positive rate is 4% (Table 2). For donor sites a 5% false negative rate (equal to 95% sensitivity) corresponds to 2.2% and 2.9% false positive rates for GenesplicerESE and GeneSplicer respectively (Table 3).

**Table 2:** False negative (FN) vs. false positive (FP) rates on test and intergenic data sets for acceptor sites

FN(%)	FP(%)			
	
	GS-test	GS-intg	GSESE-test	GSESE-intg
0.5	14.27	29.58	12.47	20.67
1	10.03	23.39	8.09	15.74
2	7.11	18.51	5.80	11.30
3	5.64	15.76	4.21	9.00
5	4.00	12.41	2.94	6.56
7	3.13	10.43	2.18	5.20
10	2.32	8.41	1.62	4.01
15	1.55	6.20	1.05	2.74
20	1.10	4.86	0.71	2.01

Rates on test data are obtained from a 5-fold CV procedure on the ESEAra data set, while FP rates on intergenic data are averages of the FP rates obtained on INTAra by setting a threshold that would produce the same FN rate on each of the 5 fold test data.

**Table 3:** False negative (FN) vs. false positive (FP) rates on test and intergenic data sets for donor sites

FN(%)	FP(%)			
	
	GS-test	GS-intg	GSESE-test	GSESE-intg
0.5	11.06	17.99	9.11	12.84
1	7.58	13.11	6.24	9.35
2	5.33	9.75	4.10	6.34
3	4.21	7.99	3.25	5.08
5	2.94	5.86	2.20	3.77
7	2.22	4.65	1.62	2.95
10	1.61	3.58	1.15	2.27
15	1.03	2.48	0.74	1.58
20	0.73	1.86	0.52	1.20

(b) Rates on test data are obtained from a 5-fold CV procedure on the ESEAra data set, while FP rates on intergenic data are averages of the FP rates obtained on INTAra by setting a threshold that would produce the same FN rate on each of the 5 fold test data.

**Figure 3:**
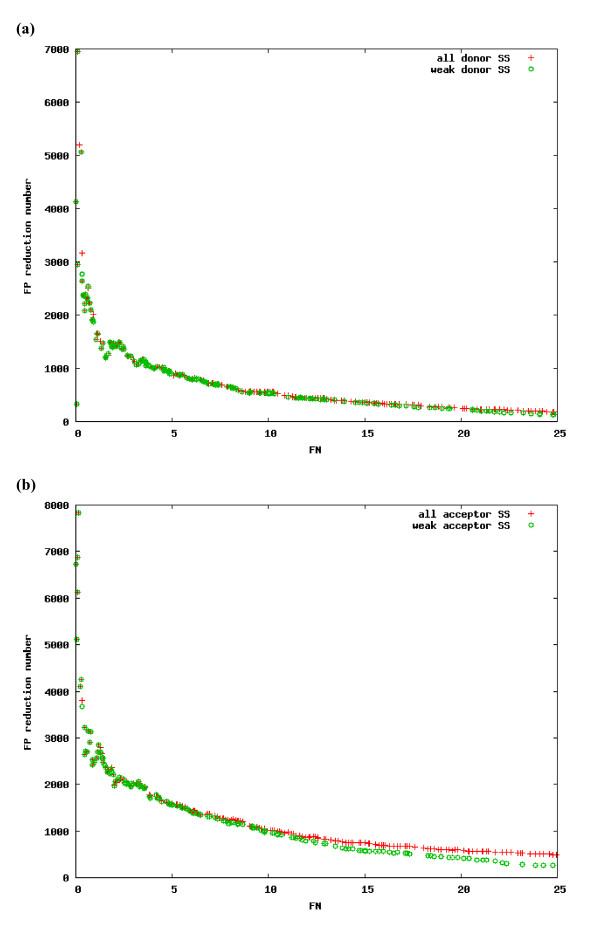
**The contribution of weak splice sites to GeneSplicerESE's performance**. For each threshold that would produce a false negative rate over all splice sites in the test data, we show the difference between the number of false positives that are predicted by GeneSplicer versus GeneSplicerESE. The red plot shows this value for all splice sites, while the green plot shows it for weak splice sites only. See Methods for definition of weak sites. (a) donor sites; (b) acceptor sites.

Since the putative ESE motifs were identified from hexamers that more frequently appear near weak splice sites than strong splice sites, it is likely that the improvement in accuracy obtained by GenesplicerESE is due primarily to an improvement in weak splice site recognition. Our results show that, with the addition of ESEs, we recover ~20% of all the weak splice sites of either type (acceptor or donor) that were missed previously (assuming a threshold of 25% false negatives). Figure 3 shows that the main contributor to GeneSplicerESE's improved prediction accuracy is its better performance on weak splice sites. Almost all of the false positives that are eliminated by use of GeneSplicerESE rather than GeneSplicer are associated with weak splice sites and this is true across a range of false negative rates.

Our experience with GeneSplicer revealed larger false positive rates on intergenic data than on sequences containing coding genes. By using our predicted ESE elements we hoped that these false positive rates could be decreased in GeneSplicerESE. Indeed GeneSplicerESE's false positive rates are significantly reduced on the INTAra data set, even more than on the ESEAra data set, probably due to the fact that the predicted ESE elements are more likely encountered into coding regions. At a threshold corresponding to a 5% false negative rate on the ESEAra data set, the acceptor sites' false positive rate for INTAra is almost twice as big in GeneSplicer vs. GeneSplicerESE (12.4% vs. 6.6%, Table 2), and significantly bigger at the donor sites (5.9% vs. 3.8%, Table 3).

Our efforts to improve splice site prediction by introducing putative ESE scores have been focused on improving our previously developed splice site predictor, GeneSplicer. The method we used here can equally well be adapted to improve other splice site prediction programs. As an example, SpliceMachineESE is a splice site predictor that we created by adding the ESE motif scores to the set of features used by SpliceMachine [29]. We downloaded SpliceMachine from the authors' website [37] and trained it using the same procedure as the one described by the original authors: a sub-sample of 1000 actual and 10000 pseudo-sites was used to obtain the optimal context sizes for all features, and then a linear SVM was trained on the complete training data set. Our training of SpliceMachine on the GSAra data set revealed false positive rates comparable to the ones previously published (ours were less than 0.1% bigger). Table 4 shows the previously reported false positive rates on the GSAra data set [29] compared to the ones we obtained for SpliceMachineESE. Even though SpliceMachine captures both positional and compositional information at all positions in large windows (at least 60 bp) around splice sites, we were still able to decrease its false positive rates (Table 4). At 95% sensitivity the false positive rate dropped from 2.1% to 1.8% for donor sites and from 2.7% to 2.4% for acceptor sites.

**Table 4:** False positive rates obtained by SpliceMachine and SpliceMachineESE on the GSAra data set

Sn	FP%			
	
	Donors		Acceptors	
	
	SpliceMachine	SpliceMachineESE	SpliceMachine	SpliceMachineESE
0.97	3.2	3.1	4.7	4.5
0.95	2.1	1.8	2.7	2.4
0.93	1.5	1.3	1.8	1.7
0.92	1.3	1.2	1.6	1.5
0.90	1.0	0.9	1.2	1.1
0.85	0.6	0.5	0.8	0.7
0.80	0.4	0.4	0.5	0.4
0.70	0.2	0.2	0.3	0.2

The false positive rates for SpliceMachine are copied from [29].

## Conclusion

In this study we identified 84 potential ESE hexamers in the flanking regions of internal coding exons from a large set of high confidence *Arabidosis thaliana *genes. These 84 ESEs were used to generate motifs with a Gibbs sampling program called ELPH. We believe these motifs to be important in splice site regulation. 35 of them have subsequently been validated experimentally to show ESE activity. We have incorporated these motifs into two splice site prediction methods and shown that they lead to an increase in accuracy for both programs.

## Methods

### Finding ESE hexamers

Many studies suggest that ESEs are present in the vicinity of splice sites. ESE activity falls off sharply with distance [38] and natural internal exons tend to be small [16]. We therefore focused our search for ESEs in the regions near the ends of exons, and we also focused on internal exons (those with introns on either side). We extracted regions of 50 bp from either end of all internal exons in the ESEAra data set, and then we identified potential ESE hexamers in these regions by using the same assumptions as the RESCUE-ESE algorithm [9]. RESCUE-ESE assumes that ESEs are represented by hexamers with both (1) a significantly higher frequency in exons than in introns and (2) a significantly higher frequency in exons with weak splice sites (also called weak exons) than in exons with strong splice sites (strong exons). To find ESEs based on these assumptions, we define "weak" splice sites as those scoring in the bottom 25% according to GeneSplicer, and "strong" splice sites as those among the top 25%. Similarly to RESCUE-ESE, we compute for each type of splice site two differences: one between the frequency of occurrence of a given hexamer *h *in exons (fEh
 MathType@MTEF@5@5@+=feaafiart1ev1aaatCvAUfKttLearuWrP9MDH5MBPbIqV92AaeXatLxBI9gBaebbnrfifHhDYfgasaacH8akY=wiFfYdH8Gipec8Eeeu0xXdbba9frFj0=OqFfea0dXdd9vqai=hGuQ8kuc9pgc9s8qqaq=dirpe0xb9q8qiLsFr0=vr0=vr0dc8meaabaqaciaacaGaaeqabaqabeGadaaakeaacqWGMbGzdaqhaaWcbaGaemyraueabaGaemiAaGgaaaaa@309A@) and the frequency of occurrence near splice sites (within 50 bp) in introns (fIh
 MathType@MTEF@5@5@+=feaafiart1ev1aaatCvAUfKttLearuWrP9MDH5MBPbIqV92AaeXatLxBI9gBaebbnrfifHhDYfgasaacH8akY=wiFfYdH8Gipec8Eeeu0xXdbba9frFj0=OqFfea0dXdd9vqai=hGuQ8kuc9pgc9s8qqaq=dirpe0xb9q8qiLsFr0=vr0=vr0dc8meaabaqaciaacaGaaeqabaqabeGadaaakeaacqWGMbGzdaqhaaWcbaGaemysaKeabaGaemiAaGgaaaaa@30A2@) and the other between the frequency of occurrence of the hexamer in weak exons (fWh
 MathType@MTEF@5@5@+=feaafiart1ev1aaatCvAUfKttLearuWrP9MDH5MBPbIqV92AaeXatLxBI9gBaebbnrfifHhDYfgasaacH8akY=wiFfYdH8Gipec8Eeeu0xXdbba9frFj0=OqFfea0dXdd9vqai=hGuQ8kuc9pgc9s8qqaq=dirpe0xb9q8qiLsFr0=vr0=vr0dc8meaabaqaciaacaGaaeqabaqabeGadaaakeaacqWGMbGzdaqhaaWcbaGaem4vaCfabaGaemiAaGgaaaaa@30BE@) and its frequency in strong exons (fSh
 MathType@MTEF@5@5@+=feaafiart1ev1aaatCvAUfKttLearuWrP9MDH5MBPbIqV92AaeXatLxBI9gBaebbnrfifHhDYfgasaacH8akY=wiFfYdH8Gipec8Eeeu0xXdbba9frFj0=OqFfea0dXdd9vqai=hGuQ8kuc9pgc9s8qqaq=dirpe0xb9q8qiLsFr0=vr0=vr0dc8meaabaqaciaacaGaaeqabaqabeGadaaakeaacqWGMbGzdaqhaaWcbaGaem4uamfabaGaemiAaGgaaaaa@30B6@). The two distributions {fEh
 MathType@MTEF@5@5@+=feaafiart1ev1aaatCvAUfKttLearuWrP9MDH5MBPbIqV92AaeXatLxBI9gBaebbnrfifHhDYfgasaacH8akY=wiFfYdH8Gipec8Eeeu0xXdbba9frFj0=OqFfea0dXdd9vqai=hGuQ8kuc9pgc9s8qqaq=dirpe0xb9q8qiLsFr0=vr0=vr0dc8meaabaqaciaacaGaaeqabaqabeGadaaakeaacqWGMbGzdaqhaaWcbaGaemyraueabaGaemiAaGgaaaaa@309A@ - fIh
 MathType@MTEF@5@5@+=feaafiart1ev1aaatCvAUfKttLearuWrP9MDH5MBPbIqV92AaeXatLxBI9gBaebbnrfifHhDYfgasaacH8akY=wiFfYdH8Gipec8Eeeu0xXdbba9frFj0=OqFfea0dXdd9vqai=hGuQ8kuc9pgc9s8qqaq=dirpe0xb9q8qiLsFr0=vr0=vr0dc8meaabaqaciaacaGaaeqabaqabeGadaaakeaacqWGMbGzdaqhaaWcbaGaemysaKeabaGaemiAaGgaaaaa@30A2@*∣h *∈ *all possible hexamers*}, and {fWh
 MathType@MTEF@5@5@+=feaafiart1ev1aaatCvAUfKttLearuWrP9MDH5MBPbIqV92AaeXatLxBI9gBaebbnrfifHhDYfgasaacH8akY=wiFfYdH8Gipec8Eeeu0xXdbba9frFj0=OqFfea0dXdd9vqai=hGuQ8kuc9pgc9s8qqaq=dirpe0xb9q8qiLsFr0=vr0=vr0dc8meaabaqaciaacaGaaeqabaqabeGadaaakeaacqWGMbGzdaqhaaWcbaGaem4vaCfabaGaemiAaGgaaaaa@30BE@ - fSh
 MathType@MTEF@5@5@+=feaafiart1ev1aaatCvAUfKttLearuWrP9MDH5MBPbIqV92AaeXatLxBI9gBaebbnrfifHhDYfgasaacH8akY=wiFfYdH8Gipec8Eeeu0xXdbba9frFj0=OqFfea0dXdd9vqai=hGuQ8kuc9pgc9s8qqaq=dirpe0xb9q8qiLsFr0=vr0=vr0dc8meaabaqaciaacaGaaeqabaqabeGadaaakeaacqWGMbGzdaqhaaWcbaGaem4uamfabaGaemiAaGgaaaaa@30B6@*∣h *∈ *all possible hexamers*} are then computed, and only those hexamers that score above a given threshold (defined in terms of standard deviations above the mean) in each of these two distributions are selected. For our A. thaliana data, we set this threshold to 1.5, which identifies ~1% of all hexamers. For other species this threshold is likely to vary, depending on the relative strength of the splice site signals.

### ELPH: Estimated-Location-of-Pattern-Hits

ELPH is a Gibbs sampling program to identify motifs present in the flanking regions of exons. Gibbs sampling has proven successful in several previous computational methods to discover motifs in regulatory sequences [39-42], although none of these previous systems focused on ESRs. ELPH takes as input a set of DNA sequences and searches through them for the most common motif. The set may contain up to several thousand sequences, and the sequences can be very short or can be thousands of nucleotides long. The algorithm's success depends on most of the sequences containing at least one copy of the motif. ELPH is freely available under an open source license from [43].

The implementation of the Gibbs sampling technique in ELPH is based on the algorithm previously described by Neuwald et al. [44]. The algorithm starts by randomly choosing a motif position in each of the input sequences. These motif positions are used to compute an initial weighted probability matrix (a position weight matrix, or pwm) describing the motif. After this initialization step, the program iteratively runs through two main steps: predictive update and sampling. In the predictive update step, one sequence from the input file is selected, beginning with the first sequence and proceeding to the last one. The motif element from that sequence is added to the background and the pwm is updated accordingly. In the sampling step, the pwm is used to assign each position in the given sequence a probability, representing the likelihood that the motif starts at that position. A motif element is assigned to the sequence by performing a weighted sample from all the possible motif positions in the sequence. These two steps are repeated until a local maximum is reached or until a pre-defined maximum number of iterations are made. The Gibbs sampler is restarted several times with different random initial conditions in order to avoid local maxima.

We ran ELPH in this fashion (as a motif detector) on the ESEAra data, looking separately at the first 50 bp (the 5' end) and the last 50 bp (the 3' end) of all exons. ELPH identified the motif TGAAGA in the 5' data and [T|C]TTC [A|C]T in the 3' data. Logos of this motifs created using WebLogo [45] are shown in Figure 1.

Another way to run ELPH is to use an input pattern as a seed. In this case the sampling step is restricted to those positions in the sequence that are close to the seed pattern. This strategy significantly constraints the search space and the output will contain the motif that best matches the input pattern.

Similar to Neuwald et al. [44], ELPH can estimate the statistical significance of any predicted motif using the Wilcoxon signed-rank test. A control set of sequences with the same background composition as the input sequences is generated using a first-order Markov model. A control sequence with the same length is appended to each sequence in the input set, and then the weighted probability matrix representing the motif is used to sample positions in the combined sequences. If the motif is a real one, then one expects the algorithm to find it in the original sequence much more often than in the random control sequence. After repeating this sampling process many times, a rank is associated to the chosen motif sites according to the frequency they have been selected. If the selected sites are from the original sequence than this rank is positive, otherwise if they fall within the control sequences the assigned rank is negative. Under the null hypothesis, the mean rank of the selected sites is expected to be zero, but largely positive if a statistically significant motif is found.

### GeneSplicerESE

Recent studies show that support vector machines [46] represent a state-of-the-art classification method for the splice site recognition task [29,47]. Based on a linear support vector machine (LSVM), we built a new splice site predictor called GeneSplicerESE. The LSVM is a binary classification technique which separates the input data points from a class *X *⊆ ℜ^*n *^by building a hyperplane with maximum distance to the closest data point from both classes (see [48] for more details). A new data point *x *∈ *X *is classified into {± 1} according to the following decision function:

*f *(*x*) = sgn (*wx *+ *b*)

where the pair {*w *∈ ℜ^*n*^, *b *∈ ℜ} describe the separating hyperplane.

GeneSplicerESE represents each candidate splice site by a feature vector consisting of the splice site score computed by GeneSplicer as described in [28], and a set of *n *motif scores computed according to the following formula:

Score(s,m)=max⁡i=1,length(s)−length(m)+1{∑j=ii+length(m)Pmj−i+1(sj)log⁡(Pmj−i+1(sj)/Pb(sj))
 MathType@MTEF@5@5@+=feaafiart1ev1aaatCvAUfKttLearuWrP9MDH5MBPbIqV92AaeXatLxBI9gBaebbnrfifHhDYfgasaacH8akY=wiFfYdH8Gipec8Eeeu0xXdbba9frFj0=OqFfea0dXdd9vqai=hGuQ8kuc9pgc9s8qqaq=dirpe0xb9q8qiLsFr0=vr0=vr0dc8meaabaqaciaacaGaaeqabaqabeGadaaakeaacqWGtbWucqWGJbWycqWGVbWBcqWGYbGCcqWGLbqzcqGGOaakcqWGZbWCcqGGSaalcqWGTbqBcqGGPaqkcqGH9aqpcyGGTbqBcqGGHbqycqGG4baEdaWgaaWcbaGaemyAaKMaeyypa0JaeGymaeJaeiilaWIaemiBaWMaemyzauMaemOBa4Maem4zaCMaemiDaqNaemiAaGMaeiikaGIaem4CamNaeiykaKIaeyOeI0IaemiBaWMaemyzauMaemOBa4Maem4zaCMaemiDaqNaemiAaGMaeiikaGIaemyBa0MaeiykaKIaey4kaSIaeGymaedabeaakiabcUha7naaqahabaGaemiuaa1aa0baaSqaaiabd2gaTbqaaiabdQgaQjabgkHiTiabdMgaPjabgUcaRiabigdaXaaaaeaacqWGQbGAcqGH9aqpcqWGPbqAaeaacqWGPbqAcqGHRaWkcqWGSbaBcqWGLbqzcqWGUbGBcqWGNbWzcqWG0baDcqWGObaAcqGGOaakcqWGTbqBcqGGPaqka0GaeyyeIuoakiabcIcaOiabdohaZnaaBaaaleaacqWGQbGAaeqaaOGaeiykaKIagiiBaWMaei4Ba8Maei4zaC2aaeWaaeaacqWGqbaudaqhaaWcbaGaemyBa0gabaGaemOAaOMaeyOeI0IaemyAaKMaey4kaSIaeGymaedaaOGaeiikaGIaem4Cam3aaSbaaSqaaiabdQgaQbqabaGccqGGPaqkcqGGVaWlcqWGqbaudaWgaaWcbaGaemOyaigabeaakiabcIcaOiabdohaZnaaBaaaleaacqWGQbGAaeqaaOGaeiykaKcacaGLOaGaayzkaaaaaa@9879@

where *s *represents a flanking region of an exon (either the 5' or 3' exonic end depending if acceptor or donor sites are classified), *m *is a motif predicted by ELPH, *S*_*j *_is the nucleotide at position *j *in sequence *s*, Pmk
 MathType@MTEF@5@5@+=feaafiart1ev1aaatCvAUfKttLearuWrP9MDH5MBPbIqV92AaeXatLxBI9gBaebbnrfifHhDYfgasaacH8akY=wiFfYdH8Gipec8Eeeu0xXdbba9frFj0=OqFfea0dXdd9vqai=hGuQ8kuc9pgc9s8qqaq=dirpe0xb9q8qiLsFr0=vr0=vr0dc8meaabaqaciaacaGaaeqabaqabeGadaaakeaacqWGqbaudaqhaaWcbaGaemyBa0gabaGaem4AaSgaaaaa@30C4@ (*a*) is the motif probability of the nucleotide *a *situated at position *k *in the motif, and *P*_*b *_(*a*) is the background probability of the nucleotide *a*. GeneSplicerESE is freely available under an open source license from [49].

## Authors' contributions

MP worked on computational identification of ESEs and designed both the ELPH and GenesplicerESE systems. SMM led the biological analysis and provided the experimental validation data for the predicted ESEs. SLS suggested the study and supervised the entire project. All authors contributed to the writing of the manuscript.

## Supplementary Material

Additional file 1 Hexamer motifs predicted as ESEs at the 5' (column 2) and 3' (column3) ends of internal exons from the ESEAra data set. Significance of the motif representation in the data (p-value) as computed by ELPH is shown for each predicted ESE, as well as an estimation of how larger is the frequency of selecting the motif in test vs. control sequences (mean rank), for 1000 sampling steps. Click here for file
